# Mining and genomic characterization of resistance to tan spot, Stagonospora nodorum blotch (SNB), and Fusarium head blight in Watkins core collection of wheat landraces

**DOI:** 10.1186/s12870-019-2093-3

**Published:** 2019-11-08

**Authors:** Jyotirmoy Halder, Jinfeng Zhang, Shaukat Ali, Jagdeep S. Sidhu, Harsimardeep S. Gill, Shyamal K. Talukder, Jonathan Kleinjan, Brent Turnipseed, Sunish K. Sehgal

**Affiliations:** 1 Department of Agronomy, Horticulture & Plant Science, South Dakota State University, Brookings, SD 57007 USA; 2 California Cooperative Rice Research Foundation, Inc., Rice Experiment Station, Biggs, CA 95917 USA

**Keywords:** Watkins landrace cultivars, Tan spot, Fusarium head blight, Stagonospora nodorum blotch, Disease resistance, Genome-wide association study, QTL, Biotic stress

## Abstract

**Background:**

In the late 1920s, A. E. Watkins collected about 7000 landrace cultivars (LCs) of bread wheat (*Triticum aestivum* L.) from 32 different countries around the world. Among which 826 LCs remain viable and could be a valuable source of superior/favorable alleles to enhance disease resistance in wheat. In the present study, a core set of 121 LCs, which captures the majority of the genetic diversity of Watkins collection, was evaluated for identifying novel sources of resistance against tan spot, Stagonospora nodorum blotch (SNB), and Fusarium Head Blight (FHB).

**Results:**

A diverse response was observed in 121 LCs for all three diseases. The majority of LCs were moderately susceptible to susceptible to tan spot Ptr race 1 (84%) and FHB (96%) whereas a large number of LCs were resistant or moderately resistant against tan spot Ptr race 5 (95%) and SNB (54%). Thirteen LCs were identified in this study could be a valuable source for multiple resistance to tan spot Ptr races 1 and 5, and SNB, and another five LCs could be a potential source for FHB resistance. GWAS analysis was carried out using disease phenotyping score and 8807 SNPs data of 118 LCs, which identified 30 significant marker-trait associations (MTAs) with -log10 (*p*-value) > 3.0. Ten, five, and five genomic regions were found to be associated with resistance to tan spot Ptr race 1, race 5, and SNB, respectively in this study. In addition to *Tsn1*, several novel genomic regions *Q.Ts1.sdsu-4BS* and *Q.Ts1.sdsu-5BS* (tan spot Ptr race 1) and *Q.Ts5.sdsu-1BL*, *Q.Ts5.sdsu-2DL*, *Q.Ts5.sdsu-3AL*, and *Q.Ts5.sdsu-6BL* (tan spot Ptr race 5) were also identified. Our results indicate that these putative genomic regions contain several genes that play an important role in plant defense mechanisms.

**Conclusion:**

Our results suggest the existence of valuable resistant alleles against leaf spot diseases in Watkins LCs. The single-nucleotide polymorphism (SNP) markers linked to the quantitative trait loci (QTLs) for tan spot and SNB resistance along with LCs harboring multiple disease resistance could be useful for future wheat breeding.

## Background

Wheat is a staple food crop for more than 35% of the world’s population [1]. Biotic and environmental stresses pose a serious threat to global wheat production [2, 3]. Fungal diseases of wheat like rusts, tan spot, Stagonospora nodorum blotch (SNB), powdery mildew and Fusarium head blight (FHB) can cause up to 50% yield losses along with a significant reduction in end-use quality [4, 5]. Further, the FHB pathogen (*Fusarium graminearum* Schwabe) produces mycotoxins such as deoxynivalenol (DON) that accumulate in the infected grains and constitute a serious threat to food safety [6]. Fungicides can be used to control these diseases to some extent, but fungicide application adds additional cost to wheat growers with inadequate control over disease like FHB [7]. Moreover, indiscriminate use of fungicides can cause environmental contamination or may lead to the development of fungal resistance.

Growing resistant cultivars is considered as an effective and eco-friendly approach to combat foliar and spike diseases in wheat. However, resistance to FHB, tan spot, and SNB is largely quantitatively inherited and limited by additive genetic effect and genotype × environment interaction [5, 8, 9]. Presently, only a couple of effective sources of resistance to FHB (*Fhb1*, *Fhb5A*) are available in cultivated bread wheat. Most of the FHB resistances have been transferred into wheat from alien species, i.e. *Leymus racemosus* (*Fhb3*), *Elymus tsukushiensis* (*Fhb6*)*,* and *Thinopyrum ponticum* (*Fhb7*) [10–12]. Currently, eight different Ptr races have been identified for tan spot [13–16], however, Ptr race 1 is found to be the most prevalent one [17–19]. Though several sources of tan spot resistance have been identified in various spring and winter wheat germplasm [9, 20–23], a greater portion of tested germplasm, including commercial cultivars, is reported to be susceptible to Ptr race 1 [9, 20, 21, 24, 25]. Similarly, SNB resistant sources also remain limited [26] and only a few commercial cultivars are known to be resistant to SNB [27]. Finally, while resistance may be derived from alien species, this type of resistance is often associated with linkage drag and may hinder progress in breeding programs. Therefore, a continuous effort in identification and introgression of resistance from under-utilized landraces can offer other alternatives to help enhance the level of resistance in modern wheat.

The success of semi-dwarf wheat varieties has resulted in large areas of wheat planted to a limited number of cultivars. While the advantages of semi-dwarf wheat are well documented, their popularity has led to limited genetic diversity and increased vulnerability to pests and diseases under the threat of changing climate [28, 29]. Previous studies showed that introgression of novel genes/alleles present in the landraces can help avert the narrowing down the genetic base of bread wheat germplasm [30, 31]. In general, the genetic diversity present in various landrace collections is much higher than in modern cultivars [32]. Therefore, mining the genetically diverse bread wheat germplasm with broad resistance to multiple diseases has the potential to improve wheat resistance to diseases and pests [33].

A. E. Watkins, a scholar from Cambridge, England, initially collected over 7000 accessions of landrace cultivars (LCs) mainly from 32 countries of Asia, Europe, Africa, and Australia in the 1930s. During the second world war, most accessions were lost, and the remaining 826 viable accessions are called Watkins collection [34]. A core set of 121 LCs was developed based on genotypic and some phenotypic evaluation that captures the majority of the genetic diversity of A.E. Watkins collection [34]. Recently, 804 accessions of Watkins collection were genotyped using a 35 K Wheat Breeders’ Array showing that a considerable amount of novel genetic diversity is present in the Watkins collection which is yet to be fully explored [35]. Several researchers evaluated the Watkins collection and found it as a potential source for identifying new genes or alleles for leaf rust, stripe rust, eyespot, and root-lesion nematode resistance [36–39]. However, these LC’s are yet to be evaluated for resistance to tan spot, SNB, and FHB.

Molecular markers linked to genes or quantitative trait loci (QTLs) can facilitate simultaneous marker-assisted breeding and pyramiding for several traits avoiding laborious and time-consuming phenotyping. Previously, QTL mapping has been used to identify marker-trait associations for *Tsr1/tsn1* [40], *Tsr2/tsn2* [23], *Tsr3/tsn3* [41], *Tsr4/tsn4* [42], *Tsr5/tsn5* [43] and *Tsr6/tsc2* [44] and three toxin sensitivity or insensitivity loci related to SNB, *Snn1* [45], *Snn2* [46], and *Tsn1* [47]. However, QTL studies have lower power in identifying QTLs with small effect and typically demarcate QTLs to large genomic regions [48], whereas the availability of high-density SNP arrays [49, 50] and next-generation sequencing technologies [51] makes genome-wide association (GWAS) a powerful tool for dissecting the genetic architecture of complex traits. Further, GWAS can effectively identify many natural allelic variations in a large set of unrelated individuals as compared to the traditional QTL mapping [52]. The effectiveness of GWAS has already been established in several crops by identifying the genomic regions controlling a variety of traits like grain shape and flowering time in rice [53, 54], husk traits [55] and stalk lodging resistance-related traits in corn [56], drought stress in barley [57], and tan spot resistance in cultivated rye [58]. In wheat, GWAS has been employed to capture genetic factors affecting complex traits like agronomic [59, 60], end-use qualities [61], and disease resistance including tan spot [62–65], Stagonospora nodorum blotch [27, 62], Fusarium head blight [66], spot blotch [67], and stem and leaf rust [68, 69]. Thus, evaluating the Watkins LCs for resistance to leaf spot and head diseases and identifying linked molecular markers through GWAS is noteworthy.

The objectives of this study were to evaluate the core set of Watkins LCs for resistance to tan spot (*P. tritici-repentis* race 1 and race 5), SNB, and FHB and identify resistant LCs that can be exploited in improving resistance to tan spot, SNB, and FHB in wheat. In addition, GWAS was performed to characterize genomic regions conferring resistance to tan spot (Ptr race 1 and race 5) and SNB in Watkins core set.

## Results

### Phenotypic/resistance evaluation

**Fig. 1:**
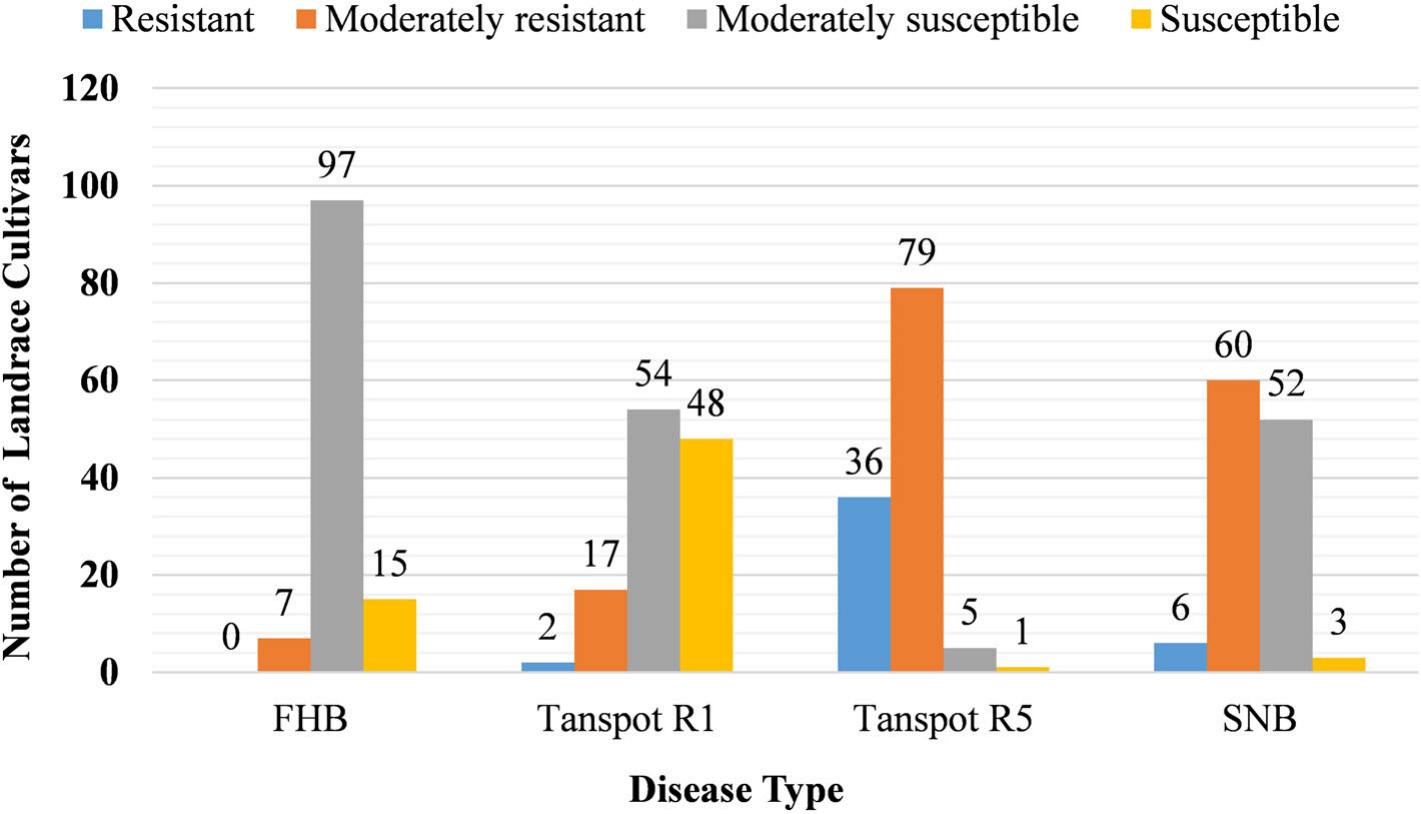
Bar graph showing the response of Watkins landrace cultivars (LCs) against Fusarium head blight (FHB), Tan spot *Pyrenophora tritici-repentis* (Ptr) race 1 (R1) and race 5 (R5) and Stagonospora nodorum blotch (SNB) evaluation. The X-axis representing the type of diseases and the Y-axis showing the number of LCs found resistant, moderately resistant, moderately susceptible, and susceptible in the evaluation. Values on the bar represent number of LCs

The Watkins core set of 121 LCs evaluated against Ptr race 1 and race 5 and corresponding toxins Ptr ToxA and Ptr ToxB respectively, showed a diverse response (Additional file 1: Table S1). Genotypic variation for both the tan spot races (Ptr race 1 and 5) were significant (*p* < 2e^− 16^) among genotypes (Additional file 1: Table S2). The mean disease scores for tan spot Ptr race 1 and Ptr race 5 among LCs were 3.6 and 1.9, respectively (Table 1). Of the 121 LCs, 2 (1.6%), 17 (14.0%), 54 (44.6%), and 48 (39.7%) were resistant, moderately resistant, moderately susceptible, and susceptible against Ptr race 1 respectively (Fig. 1). On the other hand, the majority of the LCs were found to be resistant (29.7%) or moderately resistant (65.2%) against Ptr race 5 (Fig. 1). The Pearson correlation coefficient (r) values between three repeated experiments (exp.) were 0.74 (exp. 1 and 2), 0.68 (exp. 2 and 3), and 0.75 (exp. 1 and 3) for Ptr race 1 and 0.80 (exp. 1 and 2), 0.64 (exp. 2 and 3), and 0.67 (exp. 1 and 3) for Ptr race 5.

**Table 1:** Watkins LCs found resistant/moderately resistant to leaf spot diseases and FHB

Accession No.	Country of origin	Tan spot				Stagonospora nodorum blotch (SNB)	Fusarium head blight (FHB)			
		Ptr race 1		Ptr race 5						
		Reaction type^a^ (Lesion type)	Ptr ToxA reaction	Reaction type^a^ (Lesion type)	Ptr ToxB reaction	Reaction type^a^ (Lesion type)	Accession No.	Country of origin	Reaction type^b^ (Disease Index)	Percent spikelet severity (PSS)
1190007	Australia	MR (2.28)	Insensitive	MR (1.56)	Insensitive	R (1.5)	1190032	India	MR (22.8)	10.2
1190042	France	MR (1.56)	Sensitive	R (1.22)	Insensitive	MR (1.67)	1190308	Iran	MR (23.0)	8.6
1190103	Italy	R (1.44)	Sensitive	R (1.0)	Insensitive	MR (2.61)	1190551	Spain	MR (23.75)	9.6
1190126	India	MR (2.28)	Insensitive	R (1.0)	Insensitive	MR (2.5)	1190662	Romania	MR (25.18)	9.6
1190160	Spain	MR (1.78)	Sensitive	R (1.44)	Insensitive	MR (1.56)	1190788	Turkestan	MR (25.35)	9.2
1190273	Spain	MR (2.0)	Insensitive	R (1.0)	Insensitive	MR (2.44)	–	–	–	–
1190292	Cyprus	MR (1.89)	Sensitive	R (1.11)	Insensitive	MR (1.72)	–	–	–	–
1190397	Portugal	MR (1.56)	Insensitive	R (1.17)	Insensitive	MR (2.56)	–	–	–	–
1190398	Palestine	MR (1.72)	Insensitive	R (1.22)	Insensitive	MR (2.94)	–	–	–	–
1190662	Romania	MR (2.56)	Insensitive	MR (2.0)	Insensitive	MR (2.0)	–	–	–	–
1190698	China	MR (1.83)	Insensitive	MR (1.61)	Insensitive	MR (2.17)	–	–	–	–
1190740	USSR	MR (1.67)	Insensitive	R (1.44)	Insensitive	MR (2.22)	–	–	–	–
1190912	Hungary	R (1.39)	Sensitive	R (1.33)	Insensitive	R (1.17)	–	–	–	–
Salamouni		1		1		1	Lyman		15.25	–
6B662		–		4.3		–	Overley		50	34.6
Glenlea		4.56		–		4	Emerson		–	9.1
Mean		3.6		1.9		2.8			34.1	
CV (%)^c^		12.7		19.4		14.3			14.7	
LSD^d^		0.7		0.6		0.6			9.9	
Range		1.3–4.4		1.1–4.0		1.3–4.0			17.4–56.7	

^a^Tan spot Ptr race 1 and race 5 and stagonospora nodorum blotch (SNB) disease reaction scoring from 1 to 5. ^b^Fusarium head blight (FHB) reaction type based on disease index in field experiments. ^c^*CV* Coefficient of variation ^d^*LSD* Least significant difference

A diverse response to SNB was observed among the genotypes (p < 2e^− 16^) (Additional file 1: Table S2). The mean disease score for 121 LCs was 2.8 with a range of 1.3 to 4.0 (Table 1). About 5% (*n* = 6), 49% (*n* = 60), 43% (*n* = 52), and 2.5% (*n* = 3) of LCs were found to be resistant, moderately resistant, moderately susceptible, and susceptible respectively against *P. nodorum* (Fig. 1). The Pearson correlation coefficient values between experiments were 0.76 (exp. 1 and 2), 0.69 (exp. 2 and 3), and 0.76 (exp. 1 and 3) for SNB. A variable response (p < 2e^− 10^) to FHB was also observed among the 119 LCs in the mist-irrigated, inoculated FHB nursery (Additional file 1: Table S2). The moderately resistant check Lyman showed a disease index of 15.2 and susceptible check Overley showed a disease index of 50 (Table 1). Out of 119 LCs, only seven (6%) demonstrated a moderately resistant response (DI: 13.4–25.3) while all other LCs (94%) showed moderately susceptible to susceptible (DI: 26.1–56.7) response to FHB in the field nursery (Fig. 1, Additional file 1: Table S1). In addition to FHB response, there was also a significant variation (p < 2e^− 16^) between the two replications, indicating the presence of field and inoculation variation between the replications (Additional file 1: Table S2). The mean FHB disease severity, incidence, and index in the core set were 34.4%, 98.9%, and 34.1 respectively (Table 1). The seven moderately resistant LCs were further analyzed in the greenhouse using the point inoculation method and five of these LCs displayed percent spikelet severity (PSS) ranging from 8.6–10.2% (moderately resistant), while two LCs showed moderate susceptibility (Table 1).

### Reaction of LCs to PtrToxA and PtrToxB

**Fig. 2:**
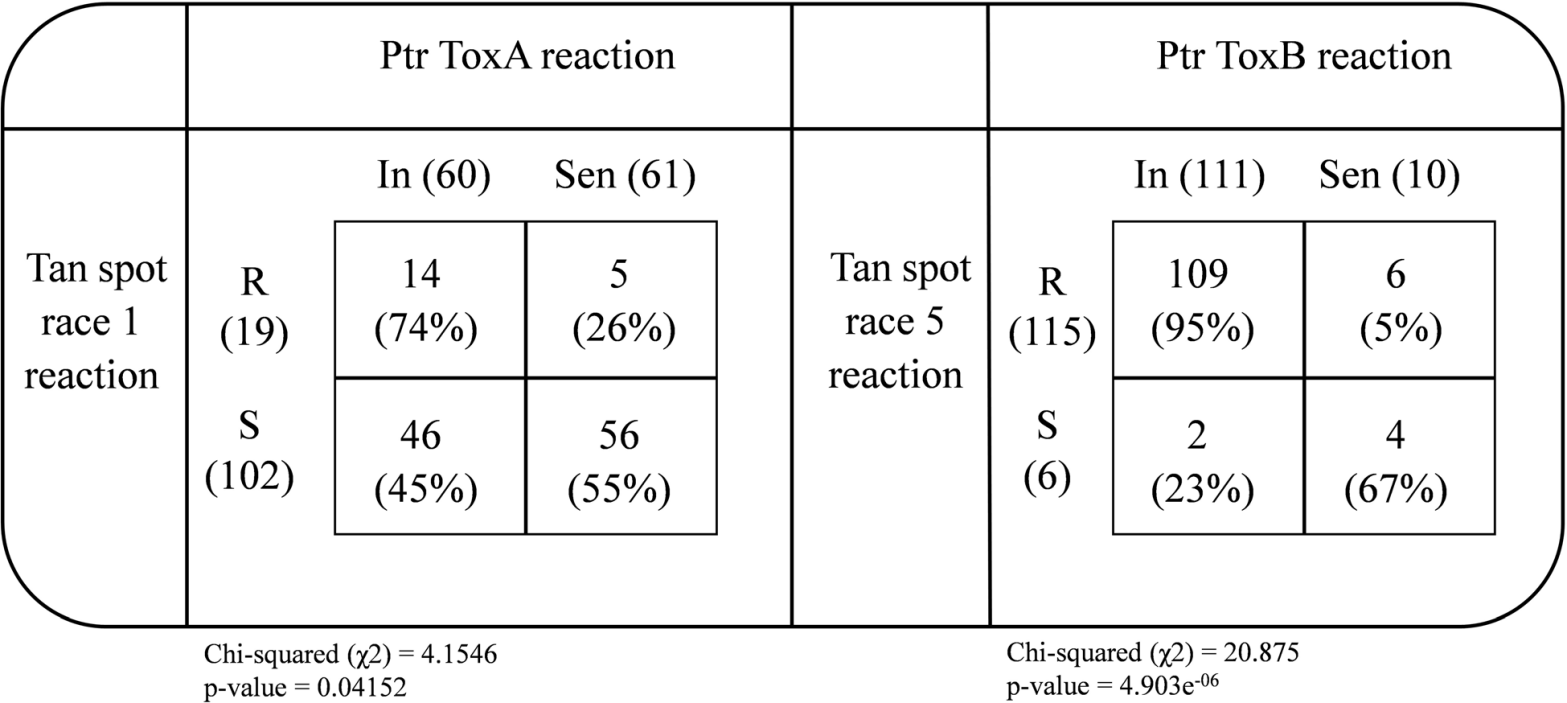
Reaction of Watkins core set of landrace cultivars (LCs) to tan spot (Ptr race 1), Ptr ToxA and Ptr race 5, and Ptr ToxB respectively. R- resistant; S- susceptible; In- insensitive to Ptr ToxA or ToxB; Sen- sensitive to Ptr ToxA or ToxB

All 121 Watkins LCs were also screened against Ptr ToxA and Ptr ToxB. Just over 50% of the LCs (*n* = 61) showed sensitivity to Ptr ToxA (produced by Ptr race 1 causing tan spot) with necrotic lesions in the toxin infiltrated leaf area, while the other 49.6% LCs (n = 60) were rated as toxin insensitive because they did not show any visible necrosis (Fig. 2). Among 19 of the resistant or moderately resistant LCs, 26% (n = 5) were sensitive and 74% (*n* = 14) were insensitive to Ptr ToxA. Out of 102 LCs that exhibited a susceptible response to Ptr race 1, 56 (55%) LCs were sensitive and 46 (45%) LCs were insensitive to Ptr ToxA (Fig. 2).

**Fig. 3:**
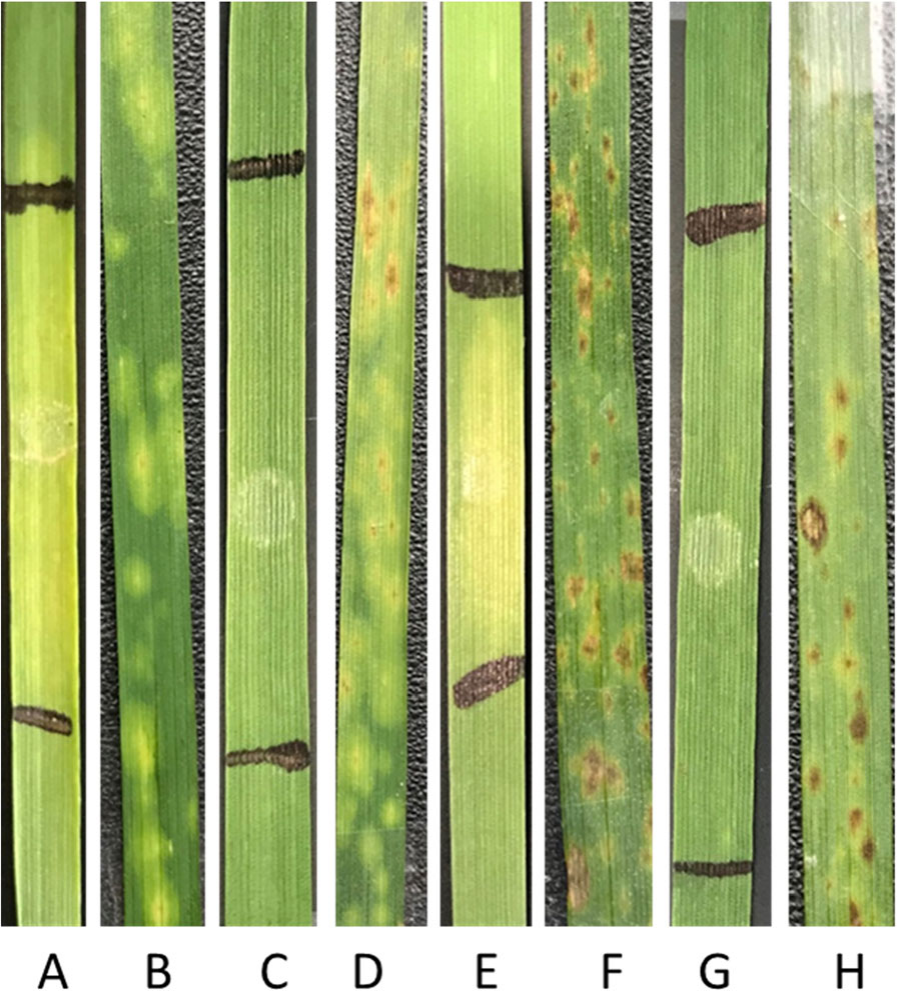
Response reaction of Watkins landrace cultivars (LCs) against *Pyrenophora tritici-repentis* (Ptr race 5) and corresponding toxin (Ptr ToxB) at the seeding stage in greenhouse. **a** Ptr ToxB reaction in 6B662 (susceptible check); **b** Ptr race 5 reaction in 6B662 (susceptible check); **c** Insensitive reaction of Acc.1190305 to Ptr ToxB; **d** Acc.1190305 showing susceptibility to race 5; **e** Acc.1190352 representing sensitivity to Ptr ToxB; **f** Acc.1190352 representing resistance to race 5; **g** Ptr ToxB reaction in Salamouni (resistant check), and **h** Ptr race 5 reaction in Salamouni (resistant check)

In case of Ptr ToxB (produced by tan spot Ptr race 5), 111 LCs (92%) displayed as insensitive with no visible chlorosis, while the only remaining 10 LCs (8%) exhibited sensitivity by producing chlorosis in the infiltrated area of the leaves. Of the 115 LCs showing resistance to Ptr race 5, 95% (*n* = 109) were insensitive to the Ptr ToxB and 5% (n = 6) were sensitive (Figs. 2 and 3). Among the six LCs susceptible to Ptr race 5, 67% (*n* = 4) and 23% (*n* = 2) manifested sensitive and insensitive response to Ptr ToxB respectively (Figs. 2 and 3). We found a significant correlation between LCs response to Ptr ToxA and Ptr race 1 (*p*-value = 0.04) and Ptr ToxB and Ptr race 5 (*p-*value = 4.903e^− 06^) (Fig. 2).

### Geographical distribution of the resistant and susceptible LCs

In this study, germplasm identified as resistant to the three diseases were collected from different parts of the world. The LCs that conferred resistance to Ptr race 1 were mainly collected from different European countries (Additional file 2: Figure S1A). On the other hand, most of the LCs resistant to Ptr race 5 were distributed around the Mediterranean Sea and southwest Asia (Additional file 2: Figure S1B). Like tan spot, the resistant or moderately resistant LCs to SNB also came from two broad geographical regions in Asia and Europe (Additional file 2: Figure S1C). Out of the five LCs moderately resistant to FHB, three were collected from Asian counties/regions (India, Iran, and Turkestan) and two from Europe (Spain and Romania) (Additional file 2: Figure S1D).

### Genotyping and population structure in Watkins core set

The 35,143 SNP genotype data for 118 LCs was obtained from Winfield et al. [35]. The data was filtered using a minor allele frequency (MAF) < 0.05 and missing value of > 10% to obtain 10,828 high-quality SNPs. Model-based Bayesian clustering of 118 LCs using 10,828 SNPs in STRUCTURE program we determined that Watkins core set was comprised of largely two main subpopulations. However, our principal component analysis (PCA) showed that 23.4% of the variation was explained by the first component (PC1), while 8.8 and 6.3% variations were explained by the second and third principal components, respectively (Additional file 1: Figure S2). Overall, a total of 38.5% of the variation was explained by the first three components. Another 2021 SNPs with no available position (cM) on the genetic map [50] were further removed to obtain 8807 SNPs that were used for GWAS analysis. Out of 8807 SNPs, 41.3% (*n* = 3639) were from A genome, 49.5% (*n* = 4356) from B genome, and 9.2% (*n* = 812) from D (Additional file 1: Table S3).

### Marker-trait associations (MTA)

**Fig. 4:**
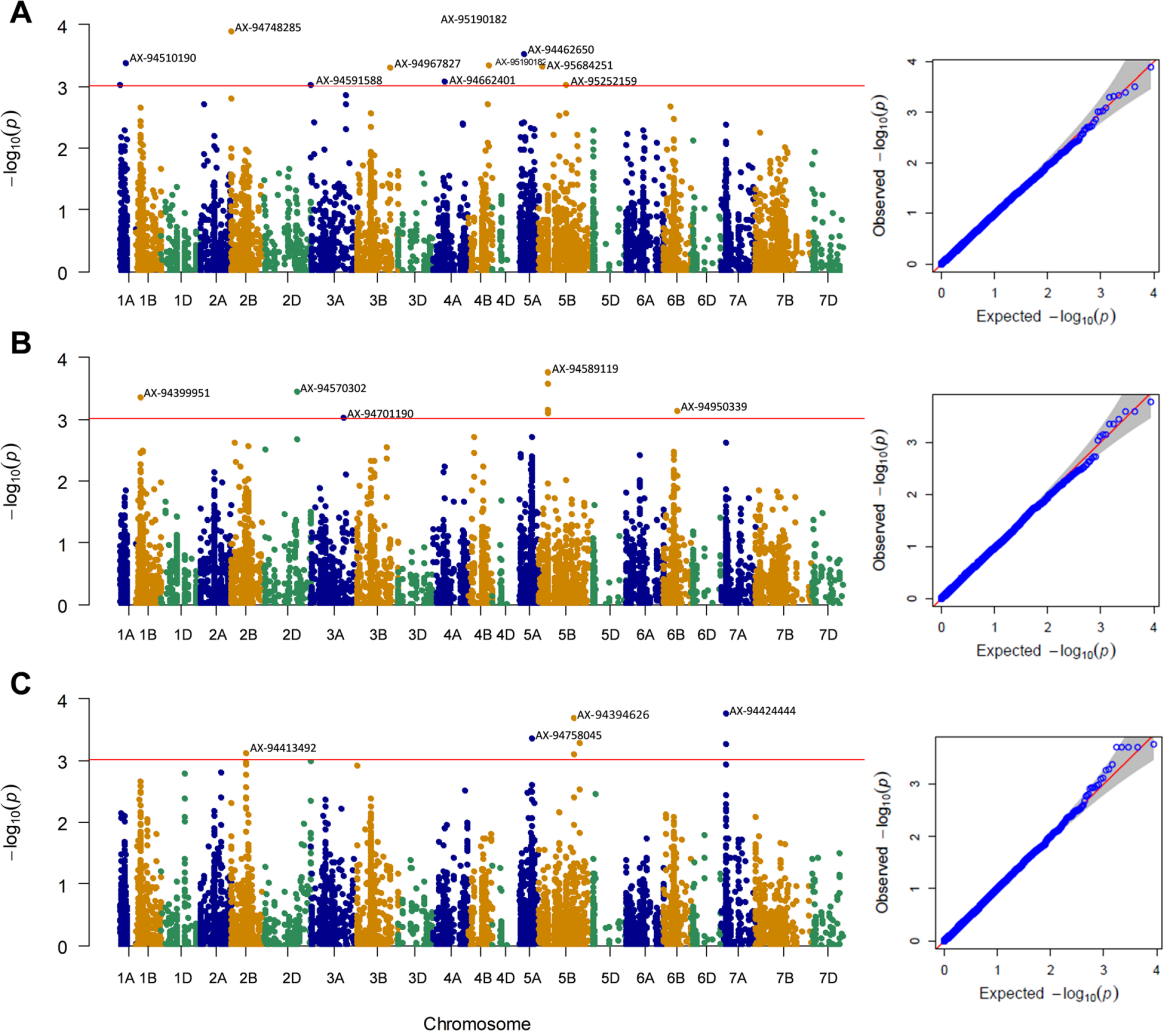
Genome-wide association scan. Mixed linear model (MLM) based Manhattan plots represent–log10 (*p-*value) for SNPs distributed across all 21 chromosomes of wheat. **a** *Pyrenophora tritici-repentis* race 1 (Ptr race 1); **b** *Pyrenophora tritici-repentis* race 5 (Ptr race 5); **c** Stagonospora nodorum blotch (SNB). Y-axis:–log10 (*p-*value) and x-axis: wheat chromosomes. The horizontal lines stand as a threshold for significant markers with–log10 (*p-*value) of > 3 which corresponds to a *p-*value < 1 × 10^− 3^. On the right side of each model, Quantile-Quantile (QQ) plots represent the expected null distribution of *p*-values vs observed *p-*values

Marker-trait associations revealed 20 putative genomic regions conferring resistance to tan spot (Ptr race 1 and 5) and SNB in the Watkins LCs of wheat (Fig. 4 and Table 2). Quantile-quantile (Q-Q) plots of *p*-values for different diseases showed that the MLM model accounting for population structure and kinship fits our data (Fig. 4). In total, 30 significant markers with -log_10_ (*p-*value) > 3.0 were identified to be associated with the traits studied. Significant markers identified 10 genomic regions associated with response to Ptr race 1 that were distributed on eight chromosomes including 1A (182.2 cM and 267.2 cM), 2B (3.1 cM), 3A (1.9 cM), 3B (202.7 cM), 4A (107.3 cM), 4B (4.99Mbp), 5A (373.0 cM), and 5B (15.7 cM and 166.7 cM). The significant markers explained phenotypic variation ranged from 14 to 17%. Five genomic regions associated with resistance to Ptr race 5 were identified on chromosomes 1B (50.4 cM), 2D (216.1 cM), 3A (198.2 cM), 5B (55.3 cM), and 6B (165.2 cM) (Table 2, Fig. 4). A QTL, *Q.Ts5.sdsu-5BS* explained the maximum variation of 20% for response to Ptr race 1. In total, six new QTLs (*Q.Ts1.sdsu-4BS*, *Q.Ts1.sdsu-5BS*, *Q.Ts5.sdsu-1BL*, *Q.Ts5.sdsu-2DL*, *Q.Ts5.sdsu-3AL*, and *Q.Ts5.sdsu-6BL*) were identified for tan spot. Association analysis for a response to SNB revealed five genomic regions on four chromosomes 2B (89.1 cM), 5A (116.6 cM), 5B (210.8 cM and 243.0 cM), and 7A (29.9 cM) (Table 2). One SNP (AX-94394626) on chromosome 5BL (*Q.Snb.sdsu-5BL*), significantly associated with SNB resistance at the seedling stage, and explained 22% of the phenotypic variation.

**Table 2:** Significant associations between single nucleotide polymorphism (SNP) markers and Watkins LCs response to two major leaf spot diseases (tan spot Ptr race 1, race 5, and SNB)

Trait	QTLs (SNP markers)	Allele	Chr	Genetic position (cM^a^)	Physical position (Mbp)	*P*-value	R ^2^
PTR1	*Q.Ts1.sdsu-1AL* (AX-94510190)	C/T	1AL	182.2	536.43	0.0004	0.16
	*Q.Ts1.sdsu-1AL* (AX-94932688)	C/T	1AL	267.2	589.02	0.0009	0.14
	*Q.Ts1.sdsu-2BS* (AX-94748285)	A/T	2BS	3.1	6.31	0.0001	0.18
	*Q.Ts1.sdsu-3AS* (AX-94591588)	C/T	3AS	1.9	20.00	0.0010	0.14
	*Q.Ts1.sdsu-3BL* (AX-94967827)	G/T	3BL	202.7	798.55	0.0005	0.16
	*Q.Ts1.sdsu-4AL* (AX-94662401)	C/T	4AL	107.3	543.74	0.0008	0.15
	*Q.Ts1.sdsu-4BS* (AX-95190182)	C/G	4BS	-^*^	4.99	0.0005	0.16
	*Q.Ts1.sdsu-5AL* (AX-94462650)	A/G	5AL	373.0	671.39	0.0003	0.16
	*Q.Ts1.sdsu-5BS* (AX-95684251)	A/C	5BS	15.7	13.43	0.0005	0.16
	*Q.Ts1.sdsu-5BL* (AX-95252159)	C/T	5BL	166.7	568.82	0.0010	0.14
PTR5	*Q.Ts5.sdsu-1BL* (AX-94399951)	C/T	1BL	50.4	352.39	0.0004	0.19
	*Q.Ts5.sdsu-2DL* (AX-94570302)	G/T	2DL	216.1	413.78	0.0004	0.19
	*Q.Ts5.sdsu-3AL* (AX-94701190)	A/G	3AL	198.2	719.76	0.0009	0.17
	*Q.Ts5.sdsu-5BL* (AX-94589119)	G/T	5BL	55.3	314.30	0.0002	0.20
	*Q.Ts5.sdsu-6BL* (AX-94950339)	C/G	6BL	165.2	678.74	0.0007	0.18
SNB	*Q.Snb.sdsu-2BS* (AX-94413492)	A/G	2BS	89.1	238.50	0.0008	0.20
	*Q.Snb.sdsu-5AL* (AX-94758045)	C/T	5AL	116.6	472.34	0.0004	0.21
	*Q.Snb.sdsu-5BL* (AX-94394626)	G/T	5BL	210.8	638.83	0.0002	0.22
	*Q.Snb.sdsu-5BL* (AX-94878132)	C/T	5BL	243.0	679.13	0.0005	0.21
	*Q.Snb.sdsu-7AS* (AX-94424444)	C/T	7AS	29.9	53.26	0.0002	0.23

^a^ The cM position is based on individual genetic maps (Allen et al. 2017) ^*^ No genetic position is available

### In silico gene annotation of the QTL regions

For response to tan spot Ptr race 1, a total of 500 genes in the 10 QTL regions with known functions in CS RefSeq v1.1 [70] were identified and 106 of those genes are predicted to have defense-related functions including major families like LRR (Leucine-rich repeat), NB-ARC (NB-ARC domain), cytochrome P450, and Pkinase (Protein kinase) (Additional file 1: Table S4). In addition, other proteins such as cysteine-rich secretory protein family (Pathogenesis-related protein 1), sugar transporter protein, peroxidase, ABC transporter, mitochondrial carrier protein, Barwin family (Pathogenesis-related protein PR-4), and acidic chitinase were found. In five candidate regions conferring resistance to tan spot Ptr race 5, a total of 207 genes identified of which only 26 known genes had a role in plant defense responses (Additional file 1: Table S4). Most of the genes belong to the protein kinase domain family. However, NBS-LRR type, NB-ARC type, and ABC transporter genes were also identified. In candidate regions conferring SNB resistance, 291 genes were identified from five QTL regions. Among them, only 36 genes were found to be associated with plant defense mechanisms. The identified proteins were mainly protein kinase domain, cytochrome P450 family, leucine-rich repeat receptor-like protein kinase family, NBS-LRR, and NB-ARC domain. (Additional file 1: Table S4).

## Discussion

Continuous improvement in wheat varieties is needed to meet the consumer demand and ensure global food security, especially with unpredictable climatic conditions causing new biotic and abiotic stresses. Mining novel resistant germplasm sources for wheat improvement could be a key breeding strategy to address these challenges. Evaluating the core set of Watkins LCs provided some useful insight about the distribution of resistant and susceptible germplasm to various diseases and identified potential LCs which could be a valuable source of resistant genes or alleles against tan spot, SNB, and FHB (Table 1).

### Geographical distribution and characterization of resistant source

A large percentage of Watkins LCs were both susceptible to Ptr race 1 and showed a resistant response to Ptr race 5. Finding resistance against Ptr race 1 is more challenging as compared to race 5 because race 1 is the most prevalent race in Africa, Asia, Europe, North and South America [13, 16, 18, 19, 71]. Other than its widespread presence, Ptr race 1 was also reported to contain the virulence of both race 2 and 3 [16], making it more aggressive than other races. In this study, most of the LCs (84%) were found to be susceptible or moderately susceptible to Ptr race 1 originated from the region around the Mediterranean Sea and all over Asia (Additional file 2: Figure S1A). This result could be partly explained by the environmental factor such as favorable weather conditions during wheat growth in the Mediterranean Sea and Asia for disease development or lower of selection pressure. Our results are in agreement with the earlier reports where a large portion of tested wheat germplasm was found susceptible to Ptr race 1 [9, 20, 21, 25, 72].

Two host-selective toxins (HST: Ptr ToxA and Ptr ToxB) produced by the various races and considered to be associated with the two symptoms necrosis and chlorosis respectively [73, 74], were used to evaluate the 121 LCs. All four combinations of toxin-disease reactions were observed among these LCs; tan spot Ptr race1 resistance-Ptr ToxA insensitive (74%), tan spot Ptr race 1 resistant-Ptr ToxA sensitive (26%), tan spot Ptr race 1 susceptible- Ptr ToxA sensitive (45%), tan spot Ptr race 1 susceptible-Ptr ToxA insensitive (55%) (Fig. 2). Data from this study support the statement that the host reaction to HST does not always determine the resistance or susceptibility of the host to Ptr races. These observations were consistent with previous studies [17, 75] and suggest that though Ptr ToxA plays a role in aggressiveness and can be used as a predictor of resistance/susceptibility, however, it is not the sole cause of pathogenicity and insensitivity to Ptr ToxA does not necessarily imply resistance to Ptr race 1 [76]. Results also suggest that other pathogenicity factors in addition to Ptr ToxA might be involved in host disease response [75, 77].

Landrace collections response to Ptr race 5 showed a majority of LCs (95%) were resistant or moderately resistant to Ptr race 5, indicating very low virulence present in this race and those lines were mainly distributed around the region of Mediterranean Sea and in southwest Asia (Additional file 2: Figure S1B). Ali et al. [21] previously reported the similar type of resistance reaction, where they found around 98% wheat genotypes resistant to Ptr race 5, however, Tadesse et al. [42] found 84% of the tested cultivars susceptible against Ptr race 5. These differences could be attributed to the different genetic backgrounds of the germplasm evaluated.

Similar to the tan spot Ptr race 1-Ptr ToxA interaction system, all four combinations of toxin-disease reactions were observed; Ptr race 5 resistance-ToxB insensitive (95%), Ptr race 5 resistant-ToxB sensitive (5%), Ptr race 5 susceptible-ToxB sensitive (23%), and Ptr race 5 susceptible-ToxB insensitive (67%) (Fig. 2). For example, accession 1190305 was insensitive to Ptr ToxB and susceptible to Ptr race 5, while, accession 1190352 was sensitive to Ptr ToxB but resistant to Ptr race 5 (Figs. 2 and 3). These four combinations of toxin-disease reaction system are fully established in Ptr race 1-ToxA interaction but the parallel relationship showing Ptr ToxB insensitivity and Ptr race 5 susceptibility observed in this study seems to be not reported so far. Therefore, results from this study suggest that germplasm which is insensitive to Ptr ToxB is not necessarily resistant to Ptr race 5 and this could be results of multiple effector-host susceptibility interactions.

Nearly half of LCs evaluated for response to SNB in this study demonstrated resistant or moderately resistant reactions, majorly dispersed in European and Asian countries, indicating that tested LCs could be a good source of resistant genes/alleles for SNB resistant wheat breeding programs (Additional file 2: Figure S1C). Several other previous studies also found around 50% of tested material was resistant or moderately resistant to SNB using both elite wheat genotypes and wheat-alien species derivatives [22, 78].

This study did not find any FHB resistant LCs within the core set of Watkins collection. However, five moderately resistant LCs that came from various parts of the world were identified. Three out of five moderately FHB resistant LCs identified in the field and greenhouse were originally collected from Asian countries/regions (India, Iran, and Turkestan), indicating Asia a potential source of resistance (Additional file 2: Figure S1D). Previous studies have shown that a high level of resistance to FHB was mainly found in Asian sources like Chinese and Japanese cultivars [4, 79]. Most (94%) of the tested LCs were susceptible or moderately susceptible to FHB, which implied that the resistant resources for FHB were rare in the Watkins collection. The five moderately resistant LCs could be further characterized and used in FHB resistance breeding.

### Marker-trait association

Ten genomic regions were identified on eight chromosomes that were significantly associated with Ptr race 1 resistance. Previous studies [40, 42, 69] have reported QTLs on eight (1AL, 2BS, 3AS, 3BL, 4AL, 5AL, and 5BL) of the 10 genomic regions, and our study supports those QTLs and identifies tightly linked SNP markers. We identified SNP AX-95252159 (*Q.Ts1.sdsu-5BL*) located on chromosome 5BL (166.7 cM), which corresponds to previous known tan spot host-selective toxin (HST) insensitivity gene *tsn1* [40, 80]. A Genome-wide association study (GWAS) was also performed on the response to toxin infiltration with a purified toxin (Ptr ToxA) that produce necrosis in leaves. Infiltration study revealed three additional SNP (AX-94912015, AX-94941069, and AX-95659861) around 150 cM on chromosome 5BL co-segregating with a genomic region very close to *Tsn1* locus [40, 80]. In addition to the known QTLs, two novel QTLs (*Q.Ts1.sdsu-4BS* and *Q.Ts1.sdsu-5BS*) on chromosome 4BS and 5BS were identified (Table 2).

Five genomic regions conferring resistance to Ptr race 5 were identified (*Q.Ts5.sdsu-1BL*, *Q.Ts5.sdsu-2DL*, *Q.Ts5.sdsu-3AL*, *Q.Ts5.sdsu-5BL*, *Q.Ts5.sdsu-6BL*) on chromosomes 1BL, 2DL, 3AL, 5BL, and 6BL (Table 2, Fig. 4). Ptr race 5 produces a toxin (Ptr ToxB) and the sensitivity to this toxin is regulated by the *Tsc2* gene which was previously mapped on the short arm of chromosomes 2B [44]. However, no significant marker-trait association on 2BS was found where the *Tsc2* gene is located. It is also likely that due to the limited statistical power, we could not detect *Tsc2* in the Watkins core set. Furthermore, previous studies related to Ptr race 5 and tan spot non-race specific studies revealed genomic regions conferring resistance on chromosomes 2AS, 4AL, and 2BL [44], 2AS and 5BL [25], 1BS and 3BL [81], 2D, 6A and 7D [63], 3B, 5D, 6B, and 7B [20]. It is clear from these independent studies that only a few common chromosomal locations have been identified related to Ptr race 5 resistance. The likely reason for rare overlap among studies could be the result of the frequency of the causal alleles in populations and small sample size. Another explanation is that the wheat-Ptr pathosystem is complex and there may be other virulence factors in addition to toxin Ptr ToxB involved in tan spot resistance [25].

Marker-trait associations for a response to SNB were identified in five genomic locations on chromosomes 2BS, 5AL, 5BL, and 7AS (Table 2, Fig. 4). Three major genes for toxin sensitivity or insensitivity, *Snn1*, *Snn2*, and *Tsn1* were previously mapped on chromosome 1BS, 2DS and 5BL, respectively [46, 47, 82]. In this study, no marker was found related to *Snn1* and *Snn2* genes. However, several markers were found co-segregating with a genomic region on chromosome 5BL where the major gene *Tsn1* is located [26]. Further, we identified a SNP significantly associated with SNB resistance on chromosome 2BS, where a resistance QTL was previously identified by Czembor et al. [83].

### In silico functional annotation of the QTL regions

Host-pathogen interaction induces a plant defense mechanism that can be divided into two major categories, (i) constitutive defense that is triggered by pathogen-associated molecular patterns (PAMPs) and (ii) a temporarily induced more localized mechanism in which plants try to defend a specific attacked area [84]. In plants, resistance (R) proteins are usually involved in pathogen recognition that triggers innate constitutive immune responses [85]. There are many R genes that have been cloned so far and most resistance proteins contain a central nucleotide-binding (NB) domain fused with a C-terminal leucine-rich repeat (LRR) domain. This study found NB-ARC and NBS-LRR type genes in many of the annotated QTL regions (Additional file 1: Table S4). The NB-ARC domain is a functional ATPase domain and its nucleotide-binding state is found to regulate the activity of R-proteins [86]. The NBS-LRR are the most common R-genes, which detect pathogen-associated proteins, typically effector molecules of pathogens that are responsible for virulence [87]. One major susceptibility gene for tan spot and SNB is *Tsn1* which encodes a protein with a leucine-rich repeat domain that is similar to the one found in NLR proteins [88]. Another large family of proteins identified in this study was Receptor-like kinases (RLKs) which is involved in various functions like plant growth, development, hormone perception and response to pathogens. Most defense-related RLKs are the LRR subclass [89]. The cloning of *Snn1* providing resistance against SNB identified Wall Associated kinases (WAKs), a unique class of receptor-like kinase (RLKs) which are known to drive pathways for biotrophic pathogen resistance. *Snn1* recognizes SnTox1, leading to activation of programmed cell death, thus allowing the necrotroph to gain nutrients and sporulate [90]. Further, we also identified peroxidase superfamily protein which is an important component of pathogen-associated molecular pattern-triggered immunity (PTI) and plays a significant role in the production of reactive oxygen species (ROS) in response to pathogen attack [91, 92]. Several other genes identified in this study are known to be related to plant defense-related responses including plant chitinase proteins that take part in pathogenesis-related activities [93], glutathione S-transferase T3 [94], serine/threonine-protein kinase [95], ABC transporter [96], pathogenesis-related protein 1(PR 1) [97], and disease resistance protein RPM1 [98].

## Conclusions

The mining of superior alleles is essential for continuous improvement in wheat germplasm. Recent diversity studies [34, 35] have shown that global collections of landraces have excellent potential. Since Watkins LCs are hexaploid wheat, like modern varieties, molecular characterization and gene introgression of useful traits could be more effective due to less linkage drag as compared to introgressions from other wild relatives. In this study, after a thorough screening of the core set of LCs against tan spot (Ptr race1 and race 5), SNB, and FHB, many potential genetic resources (Table 1) for wheat improvement were identified. This study strengthens the fact that Watkins collection is a useful genetic resource, which may confer broad resistant gene sources against various diseases [37, 38, 99, 100] and improving useful agronomic traits. As a recommendation, accession (acc.) 1190662 (Romania) could be a valuable breeding resource because it confers resistance or moderate resistance to all the diseases evaluated (tan spot Ptr race1 and race 5, SNB, and FHB) in this study. Similarly, 13 other LCs (acc.1190007, acc.1190042, acc.1190103, acc.1190126, acc.1190160, acc.1190273, acc.1190292, acc.1190397, acc.1190398, acc.1190662, acc.1190698, acc.1190740, and acc.1190912) showed resistance to tan spot (Ptr race1 and race 5) and SNB (Table 1). All these LCs could be excellent sources for current or future multi-disease resistant germplasm improvement programs. In addition, identified resistant landraces with the diverse country of origin could be a valuable source for improving the genetic diversity in wheat. Furthermore, new QTLs and tightly linked SNPs (Table 2) identified in this study may be used to develop Kompetitive allele-specific PCR (KASP) assays (Additional file 1: Table S5) for marker-assisted breeding for tan spot and SNB.

## Methods

### Plant and fungal material

A core set of 121 Watkins land race (LC) cultivars were obtained from John Innes Centre (JIC), UK [34]. The LCs used in this study were collected from more than 30 different countries in Europe, Asia, Africa, Australia, and the USSR (Union of Soviet Socialist Republics). Most of the land races were found related to two broad geographical regions. Among which 45% of the landraces come from Asian countries and 37% from Europe (Additional file 1: Table S1).

All 121 LCs were evaluated for response to tan spot caused by *P. tritici-repentis* (Ptr) race 1 (isolate Pti2) and race 5 (isolate DW7) and Stagonospora nodorum blotch (SNB) caused by *Parastagonospora nodorum* (isolate Sn2K) under greenhouse conditions at the seedling stage. A set of differential lines/cultivars Salamouni (resistant to tan spot Ptr race1, race 5, and SNB), Glenlea (susceptible to tan spot Ptr race 1 and SNB), and 6B662 (susceptible to tan spot Ptr race 5) were included as checks for tan spot and Stagonospora nodorum blotch (SNB). An aggressive *Fusarium graminarum* strain (Fg1) was used to evaluate LCs for FHB in the mist-irrigated field nursery and selected moderately resistant LCs were validated in the greenhouse. Moderately resistant cultivars Overland, Lyman, and Emerson and susceptible cultivars Flourish and Overley were used as checks for FHB.

### Evaluation of Watkin LCs for their reaction to tan spot using Ptr race 1 and race 5 and Ptr ToxA and ToxB

#### Reaction to Ptr race 1 and race 5

The core set of 121 Watkin LCs was planted in a single root trainer container (Ray Leach “Cone-trainer”™ Single Cell System) filled with Sunshine R 360 potting soil (Sun Gro Horticulture, Agawam, MA, USA). The cones were arranged in trays (Stuewe & Sons, Tangent, OR, USA) following a randomized complete block design with three replications, and the entire experiment was repeated three times. The inoculum was prepared by plating dry plugs of the isolate stored at − 20 °C in the center of petri plates containing V8PDA media (150 mL of V8 juice, 10 g of Difco PDA, 10 g of Difco agar, 3 g of calcium carbonate, and 850 mL of distilled water) [15]. V8PDA plates were wrapped with aluminum foil paper and incubated for 5–6 days at room temperature. When the culture had grown about 3 cm from the center, mycelial growth was flattened with the help of a flamed sterile test tube bottom in the presence of distilled sterilized water. Excess water was removed and the plates were incubated under continuous light for 24 h at 21 °C followed by 24 h in the dark at 16 °C to induce conidiophores and conidia, respectively. Finally, 25 mL sterile distilled water was added to each plate and the conidia were dislodged with a sterile loop wired needle. Inoculum concentration was adjusted to 3 × 10^3^ conidia mL^− 1^ using a hemacytometer. Two-week-old seedlings were spray inoculated with Ptr race 1 and 5 as described by Lamari and Bernier [15]. Following inoculation, seedlings were moved into a mist chamber to provide 100% humidity for 24 h to initiate infection. After 24 h, seedlings were transferred to a greenhouse bench at South Dakota State University, Brookings, SD. Disease response was scored 7 days after inoculation using a 1 to 5 scale lesion rating system, where scores 1–2 indicates resistant to moderately resistant, and 3–5 indicates moderately susceptible to susceptible [15].

#### Reaction to toxin Ptr ToxA and ToxB

Three fully expanded leaves of each accession were infiltrated with Ptr ToxA or Ptr ToxB culture filtrates using a needle-less syringe as described by Faris et al. [40]. Dr. Timothy Friesen, USDA-AS, Fargo, ND, kindly provided the culture filtrates. Leaves of differential genotypes such as Salamouni (insensitive to Ptr ToxA and Ptr ToxB), Glenlea (sensitive to Ptr ToxA), and 6B662 (sensitive to Ptr ToxB) were infiltrated with the equal volume (20–25 ul) of full strength filtrate. All the infiltrated plants including differential genotypes were rated after 72 h of toxin infiltration for necrosis (Ptr ToxA) or chlorosis (Ptr ToxB) symptoms and the leaves were rated as sensitive (+) or insensitive (−) reactions to each of the toxins (Ptr ToxA and Ptr ToxB).

#### Evaluation of Watkin LCs for their reaction to SNB

Seedlings were inoculated at the two-leaf stage in greenhouse using the method described for tan spot. The experiment was conducted following randomized complete block design with three replications and repeated thrice. A pure culture of Sn2k was revived on V8PDA medium by placing two dried mycelial plugs in the center of the plate. The plates were incubated at 21 °C under light for 7d. The pycnidiospores were collected by adding 30 mL sterile distilled water into each plate and by scraping the plate surface using a sterile glass slide. Inoculum concentration was estimated with a hemacytometer and adjusted to 1 × 10^6^ mL^− 1^ before inoculation. After inoculation, seedlings were moved to a humidity chamber to provide 100% humidity for 24 h and then moved back to the greenhouse bench. Disease reactions were scored 8d after inoculation using a numerical scale of 0 to 5 based on the lesion type as described in Liu et al. [45], where scores 0–2 were considered resistant and score 3 and above were considered susceptible.

### Evaluation of Watkin LCs for their reaction to FHB in field and greenhouse

#### Field evaluation

Watkins LCs along with checks were evaluated in mist-irrigated, inoculated FHB nurseries located in Brookings, SD. Each accession was planted in the field using a head-row planter in a 3-ft long row maintaining about 40 plants per row. The experiment was conducted following a randomized complete block design with two replications. Fusarium-infected corn kernels (scabby corn inoculum) were spread in the field at three, two, and one-week intervals prior to heading (beginning at boot stage). In addition, direct spray inoculation was conducted at 50% anthesis for each line using a conidial suspension containing 100,000 spores/ml and a misted irrigation was applied to maintain the humidity. Twenty-one days after inoculation, disease severity was scored for 20 spikes per LC using a visual scale described by Stack and McMullen [101]. In this scale, the percentage of the infected spikelets on each of the sampled heads were visually estimated based on 10 categories of infection (0, 7, 14, 21, 33, 50, 66, 79, 90, and 100%) and disease severity was calculated by averaging all 20 heads. Disease incidence was calculated based on the number of spikes per 20 heads showing any level of disease symptoms. Disease incidence was multiplied with disease severity to calculate the FHB disease index (DI).

#### Greenhouse evaluation

The Watkins LCs demonstrating moderately resistant responses were further evaluated in the greenhouse for Type II resistance using the point inoculation method described by Stack et al. [102]. Spore suspension was prepared from *Fusarium graminearum* (isolate Fg1) grown in ½ PDA media. The central spikelets of at least 20 spikes from each accession were inoculated at the flowering stage with l0 μl of 50,000 conidia/ml. Just after inoculation, heads were lightly misted and covered with Ziploc plastic bags to maintain the relative humidity above 90% and the greenhouse temperature was kept at 20 to 26 °C. Three days after inoculation, Ziploc plastic bags were removed. Infected spikelets of each spike were counted after twenty-one days. The total number of spikelets in each of the inoculated spikes were used to calculate the percent spikelet severity (PSS).

### Genotyping and SNP discovery

The Watkins collection was recently genotyped with the Axiom® Wheat Genotyping Breeders’ Array platform [35], which contains 35 K SNPs [50]. The genotyping data of 118 LCs were obtained from the online database CerealsDB (http://www.cerealsdb.uk.net/cerealgenomics/CerealsDB/indexNEW.php). The genotype data of 118 LCs was then filtered by removing SNPs with minor allele frequency (MAF) < 0.05 and a missing value of > 10%. The genetic positions of selected SNPs were obtained from the wheat 35 K SNP map [50]. The SNP flanking sequences were mapped using BLASTN to wheat RefSeq v1.1 assembly to identify the physical locations of the genetically mapped SNPs.

### Statistical analyses

Descriptive statistical parameters including mean, standard deviation, and coefficient of variation of disease scores (reactions) for tan spot, SNB, and FHB were calculated using R version 3.5.3 [103]. The R program was also used to perform an analysis of variance (ANOVA) to test the significance of response among LCs to different diseases. We performed Pearson’s chi-squared test to see if the toxin sensitivity/insensitivity and disease severity were correlated.

### Structure analysis

Population structure within the Watkins core set of LCs (*n* = 118) was determined by the Principal component analysis (PCA) and STRUCTURE analysis [104]. Principal component analysis (PCA) among and between the LCs was performed using the R-package ‘prcomp’. Structure analysis was done using STRUCTURE software version 2.3.4 [104] with burn-in period and a number of Markov Chain Monte Carlo (MCMC) iterations set as 10,000 and 20,000, respectively. The best-fit number of clusters (DeltaK) was determined by STRUCTURE HARVESTER [105] following Evanno et al. [106].

### Marker-trait associations (MTA)

GWAS was performed to find marker-trait association using 8807 SNP markers and the disease score data for tan spot (Ptr race 1 and race 5), and Stagonospora nodorum blotch (isolate Sn2K) with ‘GAPIT’ package [107] in the R program. Based on available genotypic information, a total of 118 LCs from the Watkins core set were used for GWAS analysis. Two linear models, the GLM (generalized linear model), which is based on the least square fixed effects and the MLM (mixed linear model), with both fixed and random effects, were evaluated. Marker effect and population structure (Q) were modeled as fixed effects, whereas the relatedness among the individuals (kinship) was modeled as random effect. A kinship matrix was calculated using GAPIT’s default VanRaden algorithm [108] and population structure (Q) was obtained using PCA [109]. The MLM method was selected for analysis because of its statistical power and ability to control type I error. Significant association of markers and traits was determined by the *p*-value < 1.0 × 10^− 3^ or -log10 (*p-*value) > 3. The MLM for GWAS can be mathematically represented as:

$$\mathrm{y}=\mathrm{X}\upbeta +\mathrm{Zu}+\mathrm{e}$$

Where, y represents the vector of the phenotypic values, β represents fixed effects due to the marker and population structure, u represents the vector of the random effects, e represents the vector of residuals, and X and Z are the incidence matrices for β and u respectively.

### Candidate gene annotation in QTL regions

The physical positions of all significant SNPs on Chinese spring (CS) RefSeq v1.1 were obtained from IWGSC [70]. To find candidate genes associated with resistance to tan spot and SNB, the candidate regions flanking the significant SNP marker were demarcated. A 5 megabase pair (Mb) region (2.5 Mb up and downstream each) from the significant SNP was selected. The CS high confidence (HC) gene annotation version 1.1 [70] was used to identify genes involved in plant defense mechanisms.

## Supplementary information

**Additional file 1: Table S1.** Watkins core set of wheat landrace cultivars, their country of origin and mean disease score with standard error for tan spot, SNB, and FHB. **Table S2.** Analysis of variance (ANOVA) response to tan spot Ptr race 1, race 5, Stagonospora nodorum blotch (SNB), and Fusarium head blight (FHB). **Table S3.** SNP distribution across the three wheat genomes used for GWAS in 121 Watkins landrace cultivars (LCs). **Table S4.** List of genes in the candidate regions spanning the tan spot race 1, 5, and SNB resistance QTLs and their functional annotations. **Table S5.** Flanking Sequence of the most significant SNP markers associated with two major leaf spot diseases (tan spot Ptr race 1, race 5, and SNB).

**Additional file 2: Figure S1.** Geographical distribution of Watkins landrace cultivars (LCs) and their response to A) tan spot Ptr race 1; B) tan spot Ptr race 5; C) SNB; and D) FHB. Red and blue spots represent resistant and susceptible LCs respectively. The figure was created using the open-source application QGIS (Version 3.8.3) and an open-source map (OpenStreetMap plugin). **Figure S2.** Principal Components Analysis (PCA) of 118 Watkins LCs of wheat. In the PCA plot, the small colored dots representing the LCs and they were colored according to three different populations (P1: Population 1, P2: Population 2, and P3: Population 3) identified by (Winfield et al. 2018) using all 804 Watkins LCs and 35 K SNPs.

## Data Availability

Supporting data and sources used for this manuscript are provided in Additional files. The genotyping data is available from Winfield et al. (2018). Seeds for the Watkins collection can be obtained from the John Innes Centre Germplasm Resource Unit (GRU http://www.jic.ac.uk/germplasm/).

## Abbreviations

ANOVA: Analysis of variance
cM: Centimorgan
DI: Disease index
DON: Deoxynivalenol
FHB: Fusarium head blight
GWAS: Genome-wide association study
IWGSC: International Wheat Genome Sequencing Consortium
KASP: Kompetitive allele-specific PCR
LCs: Landrace cultivars
MAF: Minor allele frequency
Mb: Megabase pair
MLM: Mixed linear model
MTA: Marker-trait associations
PCA: Principal component analysis
PSS: Percent spikelet severity
Ptr: *Pyrenophora tritici-repentis*
QTLs: Quantitative trait loci
RLKs: Receptor-like kinases
SNB: Stagonospora nodorum blotch
SNP: Single-nucleotide polymorphism
