# Supplementary material for: Mining and genomic characterization of resistance to tan spot, Stagonospora nodorum blotch (SNB), and Fusarium head blight in Watkins core collection of wheat landraces
Source: BMC Plant Biol. 2019 Nov 8;19:480. doi: 10.1186/s12870-019-2093-3 (PMC6839225; doi:10.1186/s12870-019-2093-3)
Supplement: Supplementary file 2 — Additional file 2: Figure S1. Geographical distribution of Watkins landrace cultivars (LCs) and their response to A) tan spot Ptr race 1; B) tan spot Ptr race 5; C) SNB; and D) FHB. Red and blue spots represent resistant and susceptible LCs respectively. The figure was created using the open-source application QGIS (Version 3.8.3) and an open-source map (OpenStreetMap plugin). Figure S2. Principal Components Analysis (PCA) of 118 Watkins LCs of wheat. In the PCA plot, the small colored dots representing the LCs and they were colored according to three different populations (P1: Population 1, P2: Population 2, and P3: Population 3) identified by (Winfield et al. 2018) using all 804 Watkins LCs and 35 K SNPs. [file 12870_2019_2093_MOESM2_ESM.docx]

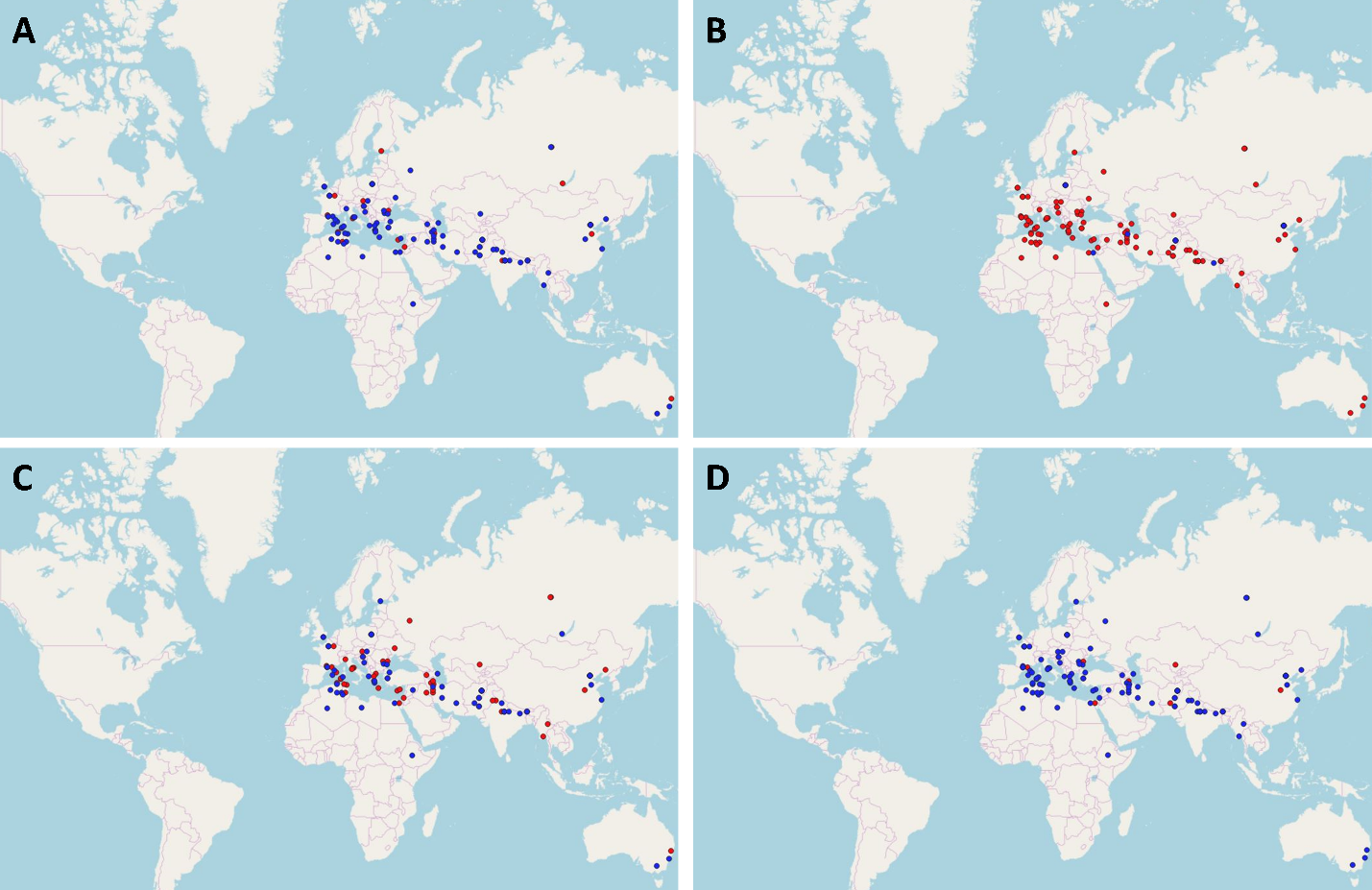


Figure S1. Geographical distribution of Watkins landrace cultivars (LCs) and their response to A) tan spot Ptr race 1; B) tan spot Ptr race 5; C) SNB; and D) FHB. Red and blue spots represent resistant and susceptible LCs respectively. The figure was created using the open-source application QGIS (Version 3.8.3) and an open-source map (OpenStreetMap plugin).

**
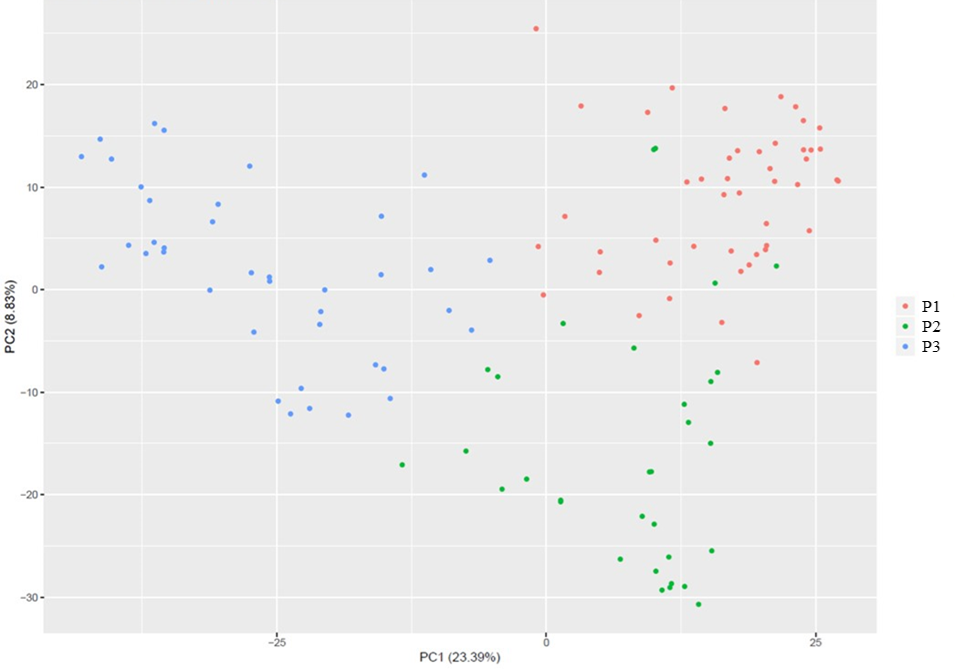
**

Figure S2: Principal Components Analysis (PCA) of 118 Watkins LCs of wheat using 10,828, SNPs. In the PCA plot, the small colored dots representing the LCs and they were colored according to three different populations (P1: Population 1, P2: Population 2, and P3: Population 3) identified by (Winfield et al 2018) using all 804 Watkins LCs and 35K SNPs.
